# Prediction model for the water jet falling point in fire extinguishing based on a GA-BP neural network

**DOI:** 10.1371/journal.pone.0221729

**Published:** 2019-09-04

**Authors:** ChaoYi Zhang, Ruirui Zhang, ZhiHui Dai, BingYang He, Yan Yao

**Affiliations:** 1 School of Technology, Beijing Forestry University, Beijing, China; 2 National Agricultural Intelligent Equipment Technology Research Center, Beijing, China; 3 Automation College, Beijing University of Posts and Telecommunications, Beijing, China; Newcastle University, UNITED KINGDOM

## Abstract

Past research on the process of extinguishing a fire typically used a traditional linear water jet falling point model and the results ignored external factors, such as environmental conditions and the status of the fire engine, even though the water jet falling point location prediction was often associated with these parameters and showed a nonlinear relationship. This paper constructed a BP (Back Propagation) neural network model. The fire gun nozzle characteristics were included as model inputs, and the water discharge point coordinates were the model outputs; thus, the model could precisely predict the water discharge point with small error and high precision to determine an accurate firing position and allow for the timely adjustment of the spray gun. To improve the slow convergence and local optimality problems of the BP neural network (BPNN), this paper further used a genetic algorithm to optimize the BPNN (GA-BPNN). The BPNN can be used to optimize the weights in the network to train them for global optimization. A genetic algorithm was introduced into the neural network approach, and the water jet landing prediction model was further improved. The simulation results showed that the prediction accuracy of the GA-BP model was better than that of the BPNN alone. The established model can accurately predict the location of the water jet, making the prediction results more useful for firefighters.

## Introduction

An analysis of the trajectory and state of the water jet is a key step in analysing the accuracy of fire extinguishing; the determination of the firing point location provides the basis for the adjustment of the relevant equipment parameters [1–3]. The location of the ignition point is the basis of water jet control, so intelligent fire-fighting equipment is the synergistic combination of ignition source location detection and jet flow control [4]. Most of the water jet control methods are based on the relationship between the ignition point and the angle of the water gun, but this approach cannot determine whether the water jet accurately falls at the ignition point [5]. The ultimate goal of firefighting water gun control is to accurately hit the ignition source point, in which case fire detection and the calculation of the ignition point space coordinates are adequate for a water sprinkler fire in the early stage of development [6]; the basic water jet state and its control situation can affect fire extinguishing, so research on the water jet is a key focus for fixed-point fire extinguishing [7].

At present, research methods on water jets in fixed-point fire extinguishing are mainly divided into two kinds: research methods that use image processing and those based on the jet trajectory equation. The research statuses of these two methods are described below.

(1) Water jet recognition and control based on image processing. Chen Jing of the Nanjing University of Aeronautics and Astronautics [8] proposed an image processing method to obtain the position of the whole water jet trajectory; her method can predict the water jet trajectory of the shelter by fitting a curve in the case of an occlusion to obtain the position of the water landing. The water cannon position can be determined by the relationship between the water shooting point and the ignition point, and then the angle of the water cannon is adjusted and revised to coincide with the fire source[9]. Sun Weilu and Zhao [10] divided water supply systems into jet and non-jet classes and they collected the pixel images by using colour clustering analysis methods [10]. The colour information was used to find the minimum RGB chromatic aberration of the current detection point in the jet direction to set the starting point for the next detection. After determining the jet direction, the ignition point was searched for in this direction. Finally, the point closest to the last image endpoint was located as the current endpoint in a suspected endpoint that satisfied the set condition. Su Hao [11] used the idea of target tracking by taking single image frames from a continuous dynamic video sequence captured by a camera as a research object to identify the water jet trajectory in each image frame and determine the water shooting location. The particle swarm algorithm [11] was used to identify the water jet trajectory against a mobile background to find and detect the target location in situations where prior information was lacking [11].

(2) Water jet research based on the trajectory equation. The fixed-point fire water flow trajectory equation is based on a classical physics equation that uses the fire water cannon parameters, for example, the working pressure of the water gun injection, work flow, sprinkler installation height and muzzle pitching angle, and external environmental factors (such as the wind speed and wind direction) to formulate the trajectory equation of the water jet. The trajectory equation is then used to predict the water flow point to achieve fixed-point fire extinguishing. Because the external factors are complex in actual situations, establishing a practical water jet trajectory equation is a critical and difficult problem. Wan Feng and other people at the Shanghai University [12–13], according to the theory of particle kinematics and external ballistics, deduced the jet motion differential equations of a water jet in air, assuming that the jet micro-element is a particle, and obtained the fitting equation by modifying the exponent on the basis of the actual jet trajectory. Liao Xiaodong and others [14] used the jet velocity as a micro-element and used it as a research object for stress analysis, water jet velocity is ejected from nozzles or orifices and diffused through the air. Through the analysis of forces in the air, the water jet trajectory equation, under the action of many influential factors, was established, and a simulation analysis of the jet trajectory of a fire gun was carried out by using MATLAB software, this method [14] overcame the problem proposed by Wan feng [12] by not constraining the working pressure and the flow parameter. Hu Guoliang and others [15] pointed out that due to a series of external factors, even if the water is injected into the space at the centre of the fire, the final flow point may exceed the scope of the margin of error for effective extinguishing. Therefore, considering gravity and the air resistance of a water flow, a force analysis was carried out. According to particle kinematics, ballistics and hydrodynamic theories, the equation of the water jet trajectory was established to model the relationships among the coordinates of each point, the height of the water cannon, muzzle pitching angle, outlet jet velocity and drag coefficient of air.

Existing methods used in water jet research have attained some achievements, but these methods have particular issues; the image processing methods require special cameras, and the environmental lighting conditions affect the results; for methods based on the jet trajectory equation, some equations need to be established under ideal conditions, and the influence of external factors (such as wind) was considered less [16–17].

With the further development and extensive application of artificial intelligence technology, the field of artificial intelligence has provided new ideas and research methods for many fields. Some studies have used artificial intelligence to study fire-fighting equipment, such as Liu Xuefei [18], who used a fire detector signal as an input to a fuzzy neural network to analyse a fire. Wang Fei [19] used the adaptive weighting algorithm to complete the data fusion of information acquired by a sensor and ignition source information.

It is very important to predict the water shooting point for effective fire extinguishing. The traditional water jet point model is a linear model, and the results ignore external factors, such as the environmental conditions and the fire engine status, which must be included considering that the prediction of the water shooting location has a complicated non-linear relationship with the firefighting equipment parameters, environmental factors and water shooting dynamics [20–21]. The BPNN has a unique advantage in dealing with nonlinear relationships and has been a widely used prediction model [22]. Therefore, this study used a BPNN to establish a model. The fire gun nozzle characteristics were included as model inputs, and the water discharge point coordinates were the model outputs [23]. The model accurately located the fire source, and allowed for the timely adjustment of the spray gun.

### Data preprocessing

According to reference [24], we selected the input variables of the water jet prediction model: the height (*h*) of the gun, the horizontal angle (*α*), the pitch angle (*β*), the pressure (*p*), the flow rate (*q*), the wind influence index (*wind*_*x*_) and (*wind*_*y*_). The output variable was the water jet landing point (*x*,*y*). Then, the parameters were trained, and some preprocessing was performed; these parameters needed to be normalized before training.

(1) Data preprocessing

Because of the index system, the measurement units of each data index were different, and the range difference was large. To have comparability among all indexes, the data were preprocessed for normalization before the weight calculation so that the data range of each index was between [0,1]. The calculation formula was as follows:

$$G_{ij}=\{\begin{array}{cc} \frac{y_{ij}-miny_{ij}}{maxy_{ij}-miny_{ij}},y_{ij}\;was & positive\\ \frac{maxy_{ij}-y_{ij}}{maxy_{ij}-miny_{ij}},y_{ij}\;was & negative \end{array}$$ (1)

(2) Determine the weights

① The weighted mean variance method

In this paper, the mean-variance weighting method was used to assign weights to each index, and the detailed calculation steps were as follows:

The mean value of each index:

$$E(G_{j})=\frac{1}{n}\sum_{i=1}^{n}y_{ij}$$ (2)

The average variance of each index:

$$\sigma (G_{j})=\sqrt{\frac{1}{n}\sum_{i=1}^{n}{\left[y_{ij}-E(G_{j})\right]}^{2}}$$ (3)

The weight coefficient of the index set:

$$W(G_{j})=\frac{\sigma (G_{j})}{\sum_{i=1}^{m}\sigma (G_{j})}$$ (4)

The result of the composite indicators was calculated as follows:

$$F(G_{j})=\sum_{j=1}^{m}W(G_{j})\cdot G_{j}$$ (5)

In addition, according to the performance description of neural network, the determinant coefficient R^2^ is calculated for the linear fitness index of the prediction system.

$$\mathrm{SST}=\sum_{i=1}^{n}(y_{i}-\bar{y}{)}^{2}$$ (6)

$$\mathrm{SSR}=\sum_{i=1}^{n}{\hat{(y}}_{i}-{\bar{y)}}^{2}$$ (7)

$$\mathrm{SSE}=\sum_{i=1}^{n}{\left(y_{i}-{\hat{y}}_{i}\right)}^{2}$$ (8)

Where, SST is the sum of total squares, SSR is the sum of regression squares and SSE is the sum of residual squares. According to the relationship between these formulas, there is SST = SSR + SSE. Therefore, the determinant coefficient is defined as:

$$R^{2}=\frac{SSR}{SST}=\frac{\sum_{i=1}^{n}{\hat{(y}}_{i}-{\bar{y)}}^{2}}{\sum_{i=1}^{n}(y_{i}-\bar{y}{)}^{2}}=1-\frac{SSE}{SST}$$ (9)

In the simulation, the "Mapminmax" function in MATLAB can realize the above calculation process to perform the normalization operation. The function maps the original data to the range of the sample set, [0,1]. The result was a structured and normalized matrix that contained the mapping relationship. In this way, when new data were obtained, it could be normalized directly without the normalization of all of the original data, and the training output results could be calculated according to the mapping information. In MATLAB, The functions of error and determinant coefficients of neural networks are defined as follows:

error = abs(T_sim—T_test)./T_test;

R2 = (N * sum(T_sim .* T_test)—sum(T_sim) * sum(T_test))^2 / ((N * sum((T_sim).^2)—(sum(T_sim))^2) * (N * sum((T_test).^2)—(sum(T_test))^2));

## Materials and methods

### Prediction model of the water jet falling point based on the BPNN

#### Construction of the prediction model based on a BPNN

**Fig 1:**
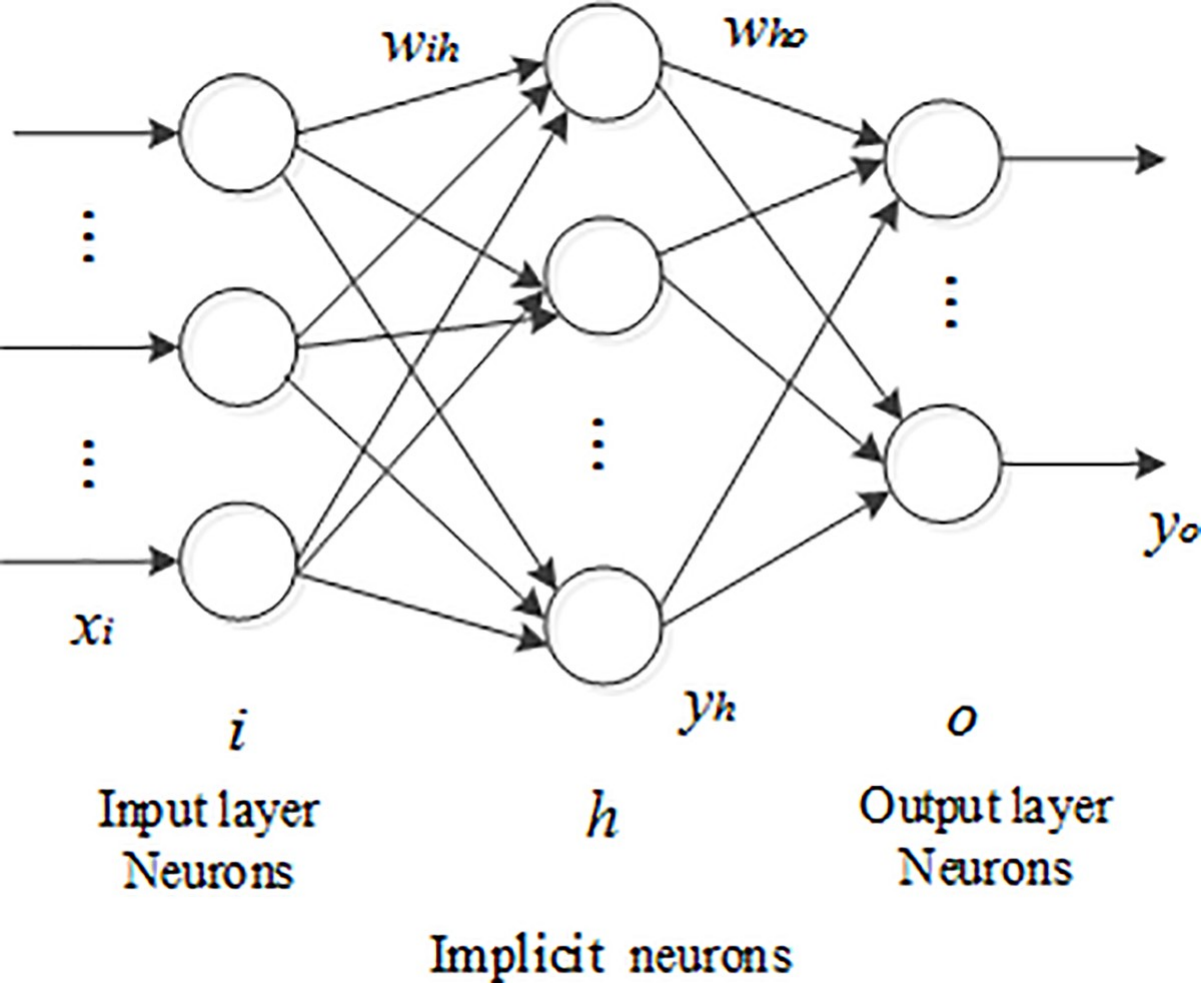
Structure of the BPNN. It is a traditional three-layer BPNN structure. By specifying the number of nodes in the input layer, hidden layer and output layer and selecting the transfer function, the hierarchical structure of the network is determined.

The fire water jet model designed in this paper was based on the BPNN. First, according to the requirements of the BPNN, the hierarchical structure of the network was determined by specifying the number of nodes in the input, hidden, and output layers and selecting the transfer functions. In terms of the number of network layers, the traditional three-layer BPNN structure, which includes an implied layer, was chosen, which is shown in (Fig 1).

In the input layer, the number of nodes was set to 7 to represent the 7 input variables of the model. The output layer had two outputs: the data type and the data value. The results of the BPNN algorithm were expressed in binary form and were used for classification and identification; the number of categories determined the number of output nodes. In this paper, the output was the coordinate value of the drop point, that is, the coordinate value of (*x*, *y*), so the number of output nodes was set to 2.

Transfer functions and training processes. In the past, there have been many studies on the transmission function of the BPNN and their deformation [25–26]. According to the previous literature and the characteristics of the water droplet drop point, the implicit layer used the hyperbolic tangent S function, which is shown in Eq (10). A linear function was selected as the transfer function of the output layer, which is shown in Eq (11). These two equations had corresponding functions in the MATLAB Toolbox library.

$$f(x)=\frac{2}{1+e^{-\alpha x}}-1\;(-1<f(x)<1)$$ (10)

f(x)=kx+c (11)

Many reference algorithms have been studied for the training of a BPNN, and different training methods have some influence on the accuracy of the prediction. From the point view of the flow impact prediction model, the LM algorithm was used to train the network, and the method for BPNN weight correction already appeared in many studies.

In this paper, the data were randomly divided into 3 sample datasets: training, confirmation and testing. The confirmation dataset was dynamically changing, and the error of each training observation decreased continuously. The error value from each training iteration was continuously monitored, and when the value was less than the threshold, the training was considered complete. After several iterations of training, the BPNN confirmation error value tended to remain steady, and the network performance was stable; if the training continued, the network's prediction results would not be much improved, so to make the error not decline further, the maximum number of BPNN training iterations was limited to avoid the "overfitting" phenomenon. The main parameters of the BPNN were set, as shown in Table 1:

**Table 1:** Neural network training parameters.

Maximum training epochs	Target error	μ	Reduction factor	Increment factor	Maximum failure limit steps
500	0.006	1	0.5	1	20

In practical applications, the number of hidden layer nodes needs to be set. If the number of nodes is too little, the network learning ability is weakened, and increasing the number of hidden layer nodes can effectively improve the learning performance. However, if the number of nodes is very large, the time complexity of network training increases, and the network training time becomes longer. Because there was no fixed method for determining the number of nodes in the hidden layer, the test method was generally used, that is, an empirical formula was used to calculate a range for the number of hidden layer nodes, and the number of hidden layer nodes was set based on the results of different experiments. In this paper, the number of hidden layer nodes was obtained using the empirical formula shown in Eq (12).

$$n=\sqrt{p+q}+a$$ (12)

where *n* is the number of hidden layer nodes, *p* is the number of input layer nodes, *q* is the number of output layer nodes, and *a* is an integer between 1 and 10. In this paper, the number of input layer neurons was set to 7 according to the inputs water gun height (*h*), horizontal angle (*α*), pitching angle (*β*), pressure (*p*), flow (*q*), wind influence index (*wind*_*x*_) and (*wind*_*y*_). The number of output layer neurons was 2, that is, the coordinate value of (*x*,*y*). According to Eq (12), the range for the number of nodes in the hidden layer was calculated as 4~13. The exact number was determined by an experimental method.

The parameters of the BPNN were set, as shown in Table 2. The number of hidden layer nodes was used to construct the network, and it is determined by the validation data, training and test datasets were used to train and test, the sample error was calculated. The mean square error of the validation data was obtained to predict the neural network performance according to the number of hidden layers nodes. The hidden layer configuration with the lowest network error was observed, and 10 neurons were selected for the hidden layer.

**Table 2:** Mean square error of the validation data with different numbers of neurons in the hidden layer.

Hidden layers	3	4	5	6	7	8	9	10	11	12
Mean square error	0.0223	0.0178	0.0127	0.0147	0.0150	0.0180	0.0127	0.0171	0.0185	0.0147
Determination Coefficient	0.18	0.14	0.16	0.15	0.13	0.16	0.14	0.15	0.13	0.16

### Experimental results

**Fig 2:**
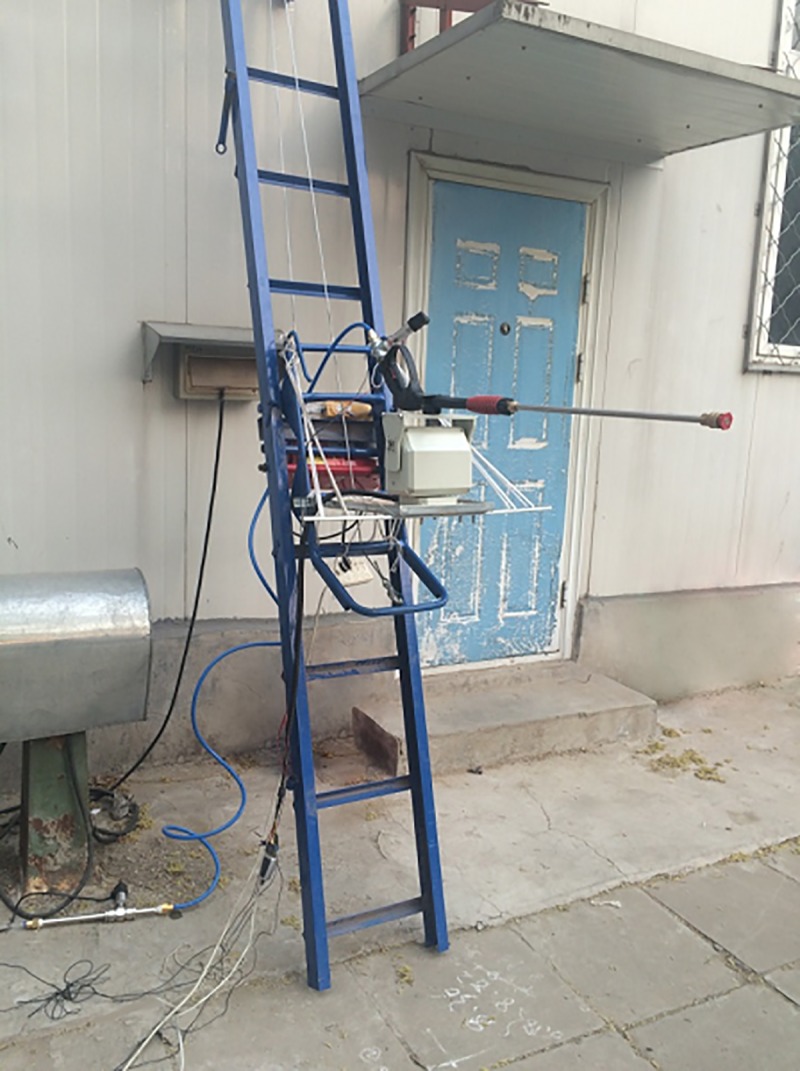
Physical chart of water jet gun experiment platform. A water jet experimental platform including automatic control water gun system was established.

In order to verify the accuracy of the method in this paper, we build a water jet experiment platform, which includes a set of automatic control water gun system, and can freely control the position of the water gun, as shown in Fig 2.

In order to change the height of water gun, the platform was fixed and placed on the elevator. The elevator can move upward and downward by controlling the positive and reverse rotation with DC motor. When it works, it can rise or fall at a uniform speed. The maximum load is about 200 kg, which satisfy the experiment requirements.

**Fig 3:**
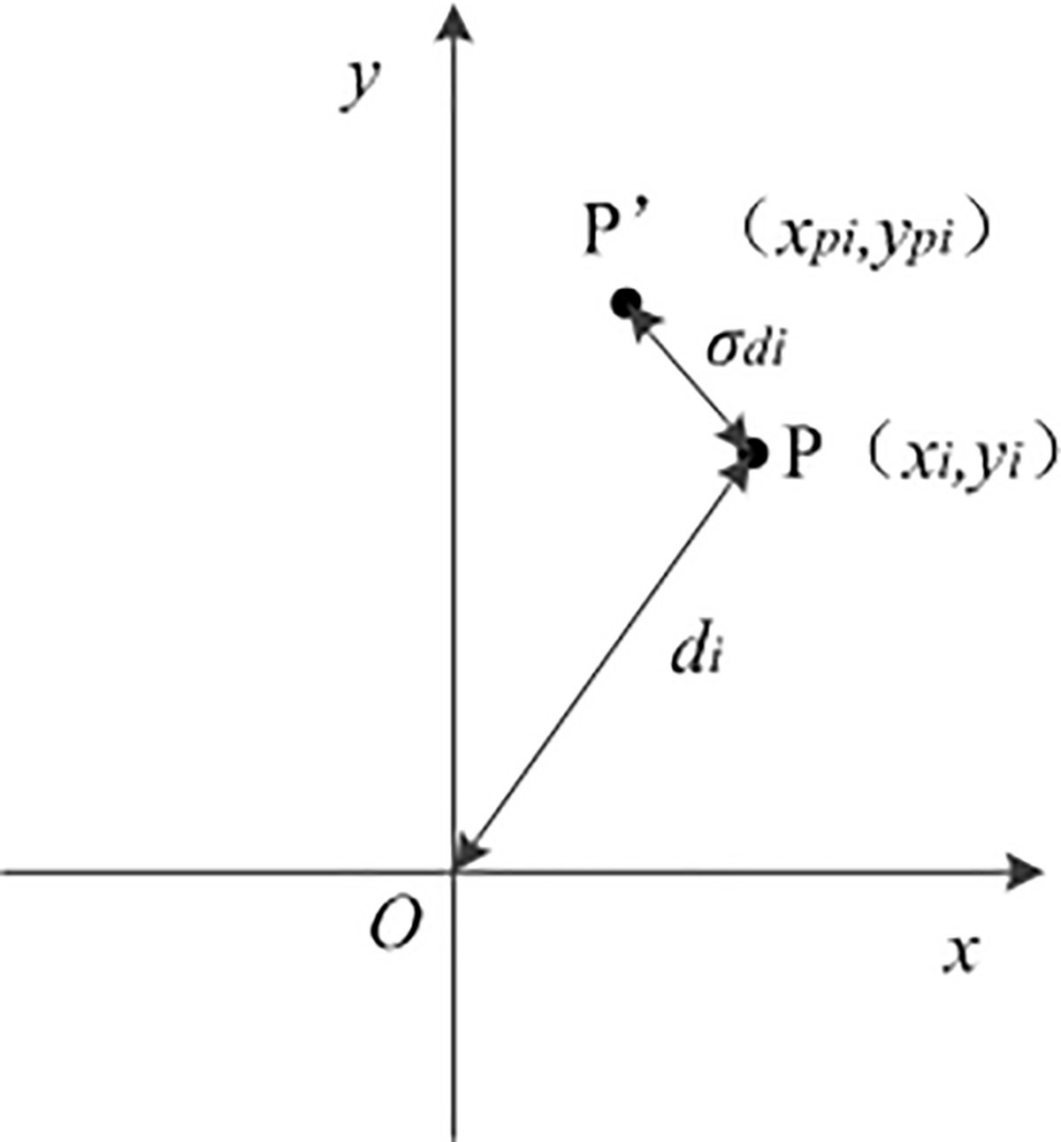
Schematic diagram of the error between the predicted and actual placements. The error schematic diagram of the experimental results and the predicted position. X is the abscissa of the water falling point, Y is the longitudinal coordinate of the water falling point. P is the landing point of water jet and P'is the deviation position.

To verify the model accuracy, it was necessary to explain the evaluation index. This paper compared the absolute and relative error results, and the experimental results of the test set are shown in Fig 3 *(Note*: *All scientific data in this paper were obtained from the author's experiments*. *They are the data of the relationship between the water jet landing point coordinates and the spray gun*, *which are primary data and have been uploaded to the server*.*)*

(1) Average distance error

The mean error was the average distance error between the predicted and actual water jet falling point, which was used (*σ*_*d*_) to express Eqs (13) and (14) as follows:

$$\sigma_{di}=\sqrt{{\left(x_{pi}-x_{i}\right)}^{2}+{\left(y_{pi}-y_{i}\right)}^{2}}$$ (13)

$$\sigma_{d}=\frac{1}{m}\overset{m}{\underset{i=1}{\sum }}\sigma_{di}=\frac{1}{m}\overset{m}{\underset{i=1}{\sum }}\sqrt{{\left(x_{p}{}_{i}-x_{i}\right)}^{2}+{\left(y_{pi}-y_{i}\right)}^{2}}$$ (14)

where σ_*di*_ is group *i* ‘s average linear distance error between the predicted and actual water jet falling point; *x*_*pi*_ and *y*_*pi*_ are the coordinates of the position of the water flow point (*p*') outputted from the BPNN for group i; *x*_*i*_ and *y*_*i*_ are the coordinates of the position of the corresponding actual water flow point (*p*) for group i; *m* is the test set sample size, which was set as 500; and *σ*_*d*_ is the average linear distance error obtained for the test dataset.

(2) Average distance relative error

The relative error = (absolute value of the measurement error) ÷ (the actual measured value). This value represented the magnitude of the error value relative to the actual measurement. The coordinates used in this paper for the water flow point are in the form (*x*,*y*). When the horizontal direction of the water flow was 0, the transverse coordinate of the position was close to 0, then the transverse relative error was large, and the prediction result was inaccurate. Here, the ratio of the water flow point distance error to the actual distance between the water shooting location and the origin was calculated as the relative error; the relative error of the water ejection point of the sample set was evaluated as the criterion, and the calculation was as follows:

$$\delta_{d}=\frac{1}{m}\overset{m}{\underset{i=1}{\sum }}\frac{\sigma_{di}}{d_{i}}\times 100\%$$ (15)

where *d*_*i*_ is the distance between the actual water jet point and the origin point for group *i* and $d_{i}=\sqrt{x_{i}{}^{2}+y_{i}{}^{2}}$.

MATLAB was used to simulate the experiments for the prediction model for the water jet displacement points of a sprinkler gun. The simulation parameters were set as follows (Table 3):

**Table 3:** Parameters of the BPNN prediction model.

Network structure	Training function	Implicit layer transfer function	Output-layer transfer function	Maximum training epochs	Target Error	μ	Reduction factor	Increment factor	Maximum failure limit steps
7-10-2	trainlm	tansig	purelin	500	0.006	1	0.5	1	20

From the data acquisition and preprocessing of the above sections, 500 data samples were obtained, and a group of 450 samples was randomly selected from the 500 samples for the neural network training sample dataset. These 450 samples are selected according to the time of water jet. Since the experiment of testing water drop point is a continuous test, the selected training and test set are based on the time of water jet gun. When we select the data of drop point, according to the continuous injection of water flow, randomly select the data in a certain period of time. 90% is the training set, that is, 90% of the time period is selected as the training sample, so that the data in the remaining 10% of the time period is used as the test set, which is more accurate. The BPNN was trained, and the remaining 30 data samples were grouped for the test sample dataset to verify the output accuracy.

Based on the abovementioned characteristics, a prediction model of the water jet falling point based on the BPNN was established. First, the results were tested 5 times with the same training set and test set, as shown in Table 4.

**Table 4:** Prediction results of the BPNN with the same test dataset.

Group	Average error distance/m	Average relative distance error	Average determinant coefficient
1	0.457	7.36%	0.23
2	0.474	6.71%	0.23
3	0.419	6.27%	0.21
4	0.438	6.82%	0.21
5	0.474	8.17%	0.23

From Table 4, it can be seen that the BPNN had large prediction deviations for the same training and test datasets. In the prediction results, the difference between the maximum and minimum errors was 0.055, and the difference between the maximum relative error and the minimum relative error was 1.9%. This was because for each training session, the initial values of the network parameters were not the same, resulting in a different starting point of the BPNN. At the same time, the convergence of the network was not the same at the end of the training session, so the network output had a large deviation.

**Fig 4:**
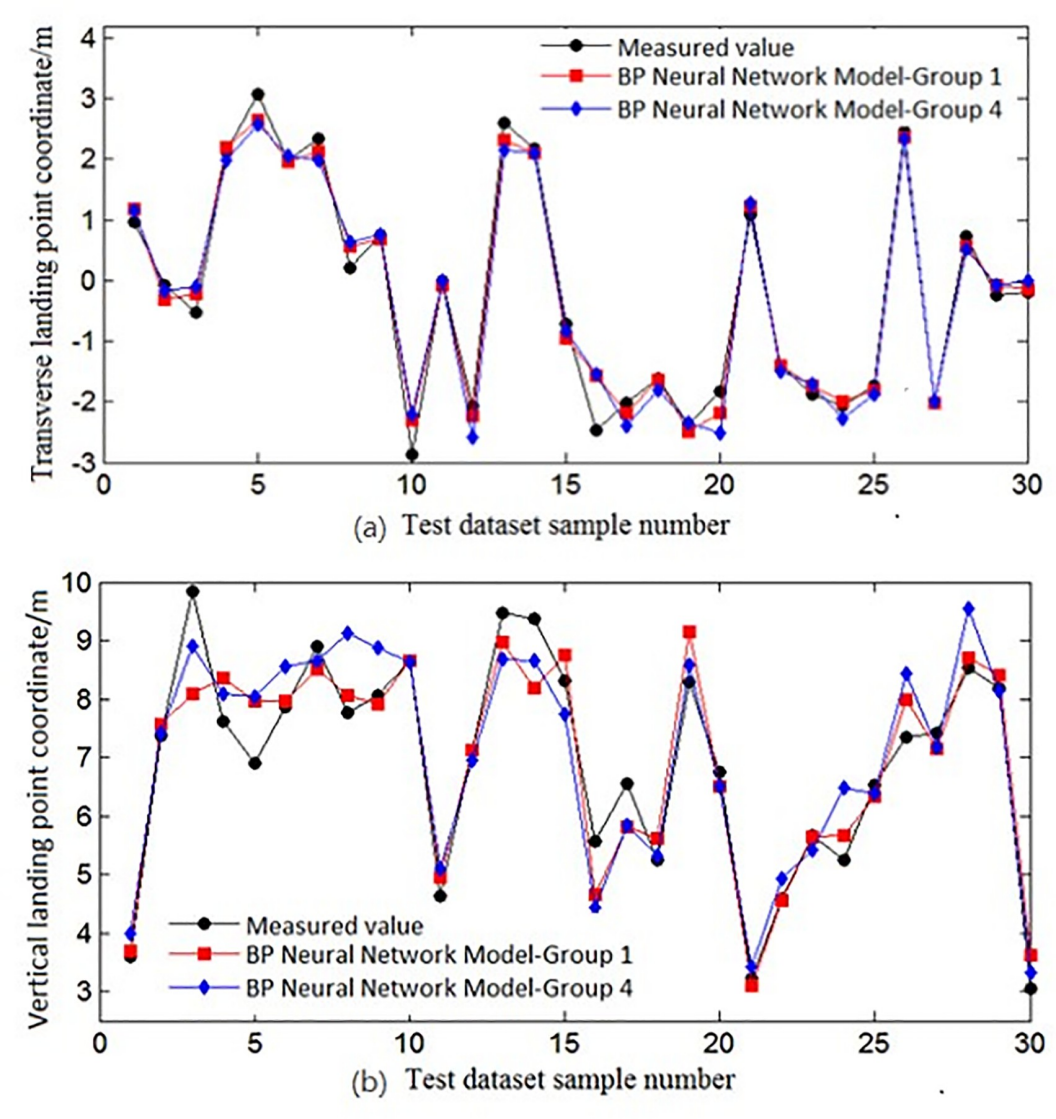
Comparison of two prediction results of the BPNN with the same data samples. The experimental results of group 1 and group 4 were compared with the predicted and measured values. (a) A comparison of coordinate values in the X direction. (b) Comparisons of coordinate values in Y direction.

To illustrate the prediction effect of the BPNN on the same sample datasets, the experimental results of groups 1 and 4 were compared with respect to the predicted and measured values, as shown in Fig 4. Groups 1 and 4 were picked out from the test dataset.

- Comparison of coordinate values in the X direction
- Comparison of coordinate values in the Y direction

It can be seen from Fig 4 that most of the predicted and actual landings were relatively close, but there were still a few instances where the water jet landings had large deviations. This also reflected the previous setting of the BPNN model weights and the initial data value settings, which influence the forecast results. It can also be seen from Fig 4 that each time the prediction result had a certain volatility, that is, the prediction model was unstable, an inherent problem was caused by the convergence of the BPNN.

### Water jet prediction model based on a GA-BPNN

#### Genetic algorithm optimization of the BPNN

Literature on the optimization of BPNNs based on genetic algorithms [27–28] is extensive. The GA algorithm can improve the BPNN's slow convergence, which easily falls into the local optima and other issues [25][29]. The optimization goals were the optimization of network weights, the network structure, and learning rules [26][30]. In this paper, the weights and thresholds were optimized by a genetic algorithm. The genetic algorithm mainly included several coding design steps for the individual fitness evaluation, genetic operators, and main operating parameters. If these parameters were determined, the BPNN can use the algorithm to match and optimize the weights in the network so that it can be trained to obtain the global optimization state [27–28][31–32].

#### Construction of the GA-BP neural network prediction model

In the previous section, the principle of the genetic algorithm optimization of a BPNN was briefly introduced. Next, the construction of the BPNN water jet prediction model based on genetic algorithm optimization (hereafter referred to as the GA-BPNN prediction model) will be specifically described.

**Fig 5:**
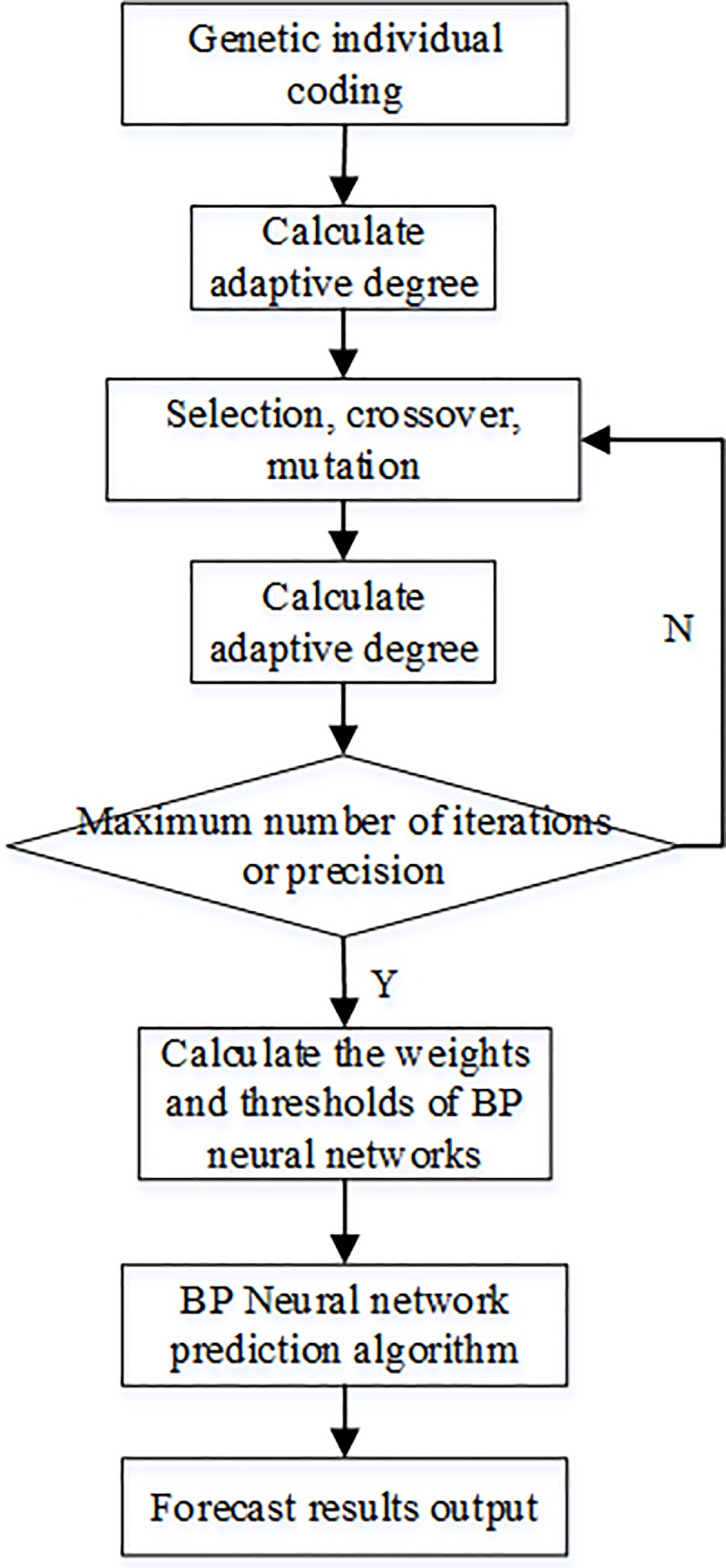
Flow chart of the GA-BP neutral network prediction algorithm. The work chart of BPNN water jet prediction model based on genetic algorithm optimization was constructed.

There have been many previous studies on using genetic algorithms for BPNNs. We used this model to predict the water jet landing point model. The workflow is shown in (Fig 5):

The algorithm for optimizing the weights of a BPNN using a genetic algorithm is as follows:

The first step was the individual coding and population initialization. In the BPNN, the weights of each neuron were arranged according to certain rules, and the individuals were generated. An encoding process was used to obtain the individuals in a random manner. This process was actually the process used to generate the initial population of a genetic algorithm. This article adopted a real number coding method to encode the individuals, assuming that the chromosome length was S, that is, the encoding length, as seen in Eq (16).

S=p×n+n×q+n+q (16)

where *p*, *n* and *q* were introduced earlier and the initial values were given. After the calculation, S = 116. When solving for the global optimal solution of the neural network, the larger the population, the easier it was to find an optimal solution, but the computational complexity would also increase. Therefore, the number of populations was set to 80 in this paper.

The second step consists of the adaptive degree calculation. The fitness function is shown in Eq (17).

$$F=\frac{1}{E}$$ (17)

where *E* is the mean square error of the output prediction result and the actual expectation. The value reflects the probability of individual survival. The higher the value, the more accurate the output prediction result is.

The third step consists of the selection of individuals. In the genetic algorithm, the principle of individual selection is to select the best individuals for producing the next generation from the existing population and address the convergence problem of an optimal individual, i.e., a local optimal solution was found. In this paper, the individuals were selected by using the roulette selection method. The probability of selection is shown in Eq (18):

$$P_{i}=\frac{f_{i}}{\sum_{k=1}^{s}f_{k}}\;(i=1,2,\ldots ,s)$$ (18)

where *f*_*i*_ is the individual *i* ‘s fitness value in the population and *S* is the number of individuals in the population. To achieve the best fitness for individuals in the subsequent crossover and mutation operations, the best-preserved individual strategy was used, i.e., the individuals with the best fitness were replaced by the individuals with the worst fitness in this iteration.

The fourth step consists of the crossover operation. The crossover operation in the genetic algorithm is related to the coding method. It is a step of acquiring a new individual through the exchange of the parental genomic chromosomes, which is determined by the crossover probability. This article used the real-coded methods, as shown by Eqs (19) and (20):

$$X_{A}^{i}{}^{'}={\mathrm{\alpha X}}_{B}^{i}+(1-\alpha )X_{A}^{i}$$ (19)

$$X_{B}^{i}{}^{'}={\mathrm{\alpha X}}_{A}^{i}+(1-\alpha )X_{B}^{i}$$ (20)

where α is a random number between [0–1]; *X*_*A*_ and *X*_*B*_ are a pair of chromosomes chosen at random for the crossover operation; $X_{A}^{i}$ and $X_{B}^{i}$ are the corresponding gene positions for *X*_*A*_ and *X*_*B*_, respectively; *i*∈[1,S] (S is the length of the chromosome), and XAi' and XBi' are the new genes that produced by the crossover operation [33].

The fifth step involves the mutation operation. In a GA, individual variation is the cause of population diversity. By using the mutation operation, the GA can avoid the premature convergence of the neural network. This article used Eq (21) for the mutation operation. Through the non-uniform mutation operator, the iteration step size can be adjusted according to different generations so that the convergence range was further reduced compared with that of the BPNN.

$$\{\begin{array}{l} X_{A}^{i}{}^{'}=X_{A}^{i}+\left(U_{\max}-X^{i}\right)\times \gamma \;\beta \geq 0.5\\ X_{A}^{i}{}^{'}=X_{A}^{i}-\left(X^{i}-U_{\min}\right)\times \gamma \;\beta <0.5 \end{array}$$ (21)

where $\gamma ={\left[r\times \left(1-\frac{t}{t_{\max}}\right)\right]}^{2}$, *r* is a random number from 0 to 1, *t* is the evolutionary algebra, *t*_*max*_ is the set maximum number of generations, *β*<1, *X*^*i*^ is the gene at the mutation position of the mutation chromosome, X^i^∈[U_min_, U_max_], and *X*^*i*'^ is the new gene produced by the mutation operation.

The sixth step is to cycle and terminate. Determining the termination of the genetic algorithm mainly depends on whether an iteration has reached the requirements. If none of the termination conditions are satisfied, the cycle is continued for steps 2~5.

Finally, after terminating the genetic algorithm, the optimal individual results obtained were used in the BPNN; that is, the weights on each layer were re-evaluated, and the subsequent steps were performed, such as training. In this way, the predictions were made, and the model outputs were closer to the actual water jet drop point locations.

## Results and discussion

This section covers the verification of the simulation and the analysis of the abovementioned algorithm. The water jet prediction model based on a GA-BPNN is constructed and is compared to the water jet prediction model using the traditional BPNN. The parameter setting part of the GA-BPNN was consistent with that of the BP network in the previous section. In addition, the GA-BPNN genetic algorithm parameter settings are shown in Table 5.

**Table 5:** Parameters of the genetic algorithm.

Parameters	Value	Instructions
Population size	80	Total number of individuals per generation
Crossover probability	0.25	The probability of the two paired chromosome gene exchanges in the parent
Variation probability	0.01	The probability of a chromosomal gene mutation in the father
The largest evolutionary generation	10	The maximum number of iterations of the genetic algorithm; one of the evolutionary stop conditions
Maximum continuous evolutionary algebra	5	One of the evolutionary stop conditions

The same data were used as with the BPNN simulation experiments in the previous section, this article used the same experiment to predict the falling point of water flow. Here, the GA-BPNN algorithm was first used to preprocess the data, and the same training and sample datasets were processed and operated upon. The experiment was performed 4 times, as shown in Table 6.

**Table 6:** Prediction results of the GA-BPNN with the test dataset.

Group	Average error distance	Average relative error	Average determinant coefficient
1	0.301	4.34%	0.15
2	0.310	4.68%	0.15
3	0.396	4.51%	0.18
4	0.304	4.47%	0.15

From Table 6, we can see that for the same dataset, using the GA-BPNN prediction results, the differences were very small for the prediction accuracies, the average relative error of the maximum and minimum difference was 0.34%, and the error range was 0.14 m. The results indicate the performance relative to that using the BPNNs alone, especially in terms of the ability of local convergence in GA, so that each iteration will choose the best individuals to pass on from one generation to the next. Through the optimization of the weights and thresholds between the neural network layers, the prediction results were achieved more accurately, and the prediction differences were further reduced.

To further compare the results of the GA-BPNN and BPNN for the prediction of the water jet falling point, the following four sets of training and test datasets were selected for experimentation. Ten predictions were performed separately, and the average of the 10 errors was taken as the prediction result. The data results were recorded, as shown in Table 7.

**Table 7:** Comparison of prediction results for the BP and GA-BP models.

Group	BPNN prediction model		GA-BPNN prediction model	
	Average error distance	Average relative error	Average error distance	Average relative error
1	0.471	7.59%	0.317	4.59%
2	0.485	7.25%	0.337	4.96%
3	0.507	7.62%	0.343	4.74%
4	0.491	7.36%	0.345	4.66%

An output result error always exists. This was because the prediction process of the neural network itself had errors, and the inaccuracies in the actual measurement and the settings of the experimental simulation environment will also bring about certain errors. To further eliminate the influence of these accidental errors, this experiment focused on different data and performed multiple sets of measurements and simulations on the two predictive models. From Table 7, we can see that the predictions from the model based on the GA-BPNN were better than those of the BPNN model in terms of distance error and relative error.

**Fig 6:**
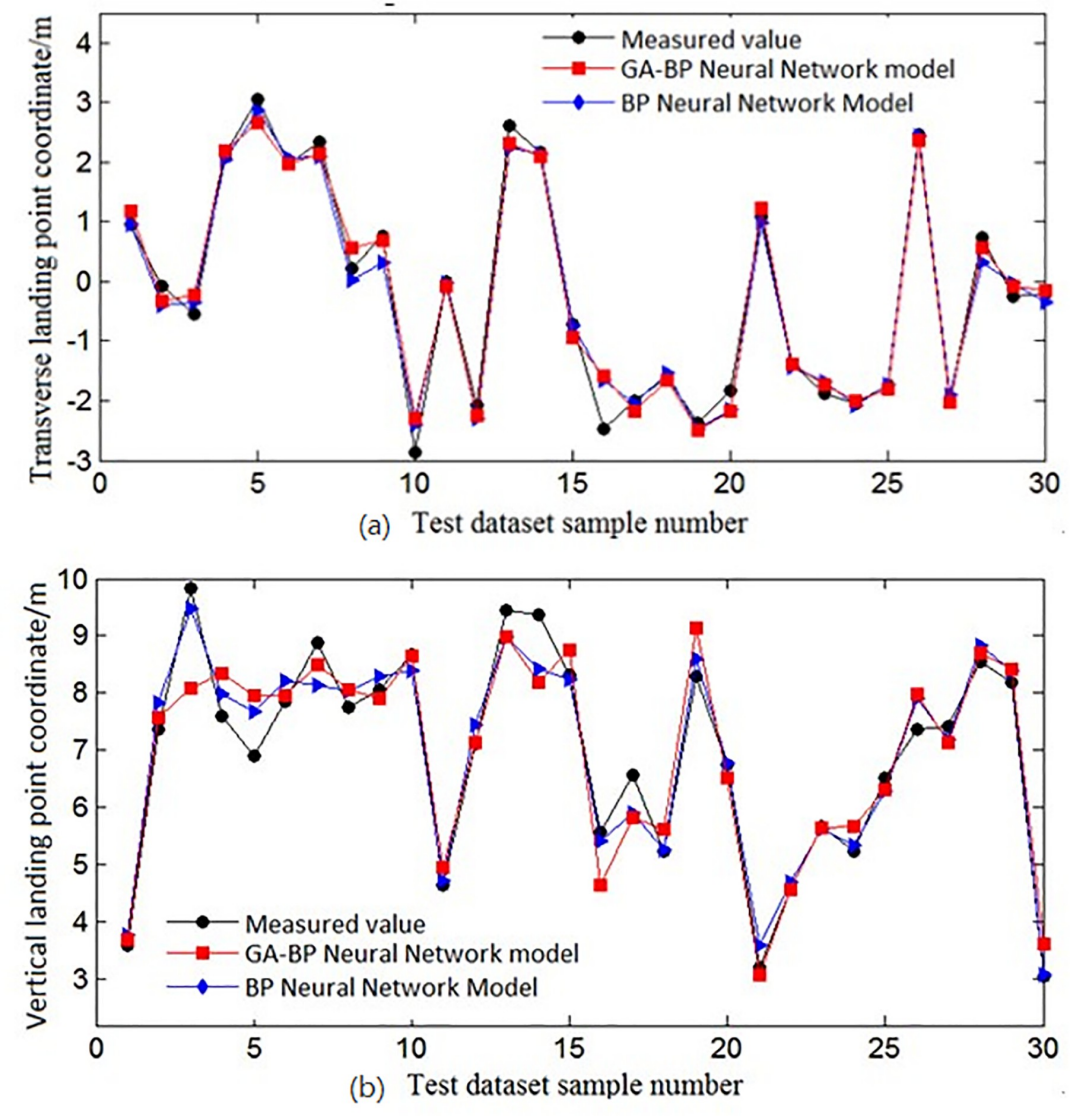
Comparison of the predicted values and measured values of the two predictions. The best experimental data are used as comparison, and the same sample set and training set used by the GA-BP and BPNNS were used for prediction modeling. (a) A comparison of coordinate values in the X direction. (b) A comparison of coordinate values in the Y direction.

The above experimental schematic diagram of the graphical results is given below, as shown in Fig 6. For the different groups, the same sample set and training set used by the GA-BP and BPNNs were used for predictive modelling. A total of 20 experiments were performed. The best experiment was used as a comparison to observe the two networks’ predicted and measured values.

- Comparison of the coordinate values in the X direction
- Comparison of the coordinate values in the Y direction

It can be seen from Fig 6 that due to multiple sets of experiments and taking the data from the experiment with the best results as the comparison, the actual error in these two algorithms was not large. However, it still can be seen that for the predictions based on the GA-BP, the predicted water jet landing point was basically consistent with the actual landing point. Compared with the BPNN prediction, the error appeared to be relatively large, and the difference between these two algorithms is reflected in Table 7.

From the comparison between Tables 6 and 7, it can be seen that the BPNN was inferior to the GA-BPNN in terms of local convergence, and there was some deviation in the final prediction. This deviation will be judged by firefighters in an actual fire extinguishing process.

## Conclusion

This paper used the classical BPNN to model and predict the water jet falling point of a fire gun and designed a network structure suitable for the fire water jet landing point model, including the data collection, data preprocessing, network weight setting, transfer function selection and other parameter settings. Then, experiments and predictions were carried out. As a result, it was found that the prediction error of the water jet landing point based on the BPNN was relatively large. Then, a water jet prediction model based on the GA-BPNN was proposed. The article adopted the optimization structure of the genetic algorithm to analyse and optimize the neural network weights and thresholds, and, to a large extent, address the problems of the BPNN training process in terms of local minima and other issues. The simulation results showed that the accuracy of the GA-BP model prediction was better than that of the BPNN. The established model can accurately predict the location of the water jet, making the prediction result more useful for firefighters.

## Supporting information

S1 File Structure of experiment system. (PDF)

S2 File Water jet coordinate data set. (RAR)

S3 File Platform experiment data of water jet system. (PDF)
