# Supplementary material for: Prediction model for the water jet falling point in fire extinguishing based on a GA-BP neural network
Source: PLoS One. 2019 Sep 4;14(9):e0221729. doi: 10.1371/journal.pone.0221729 (PMC6726229; doi:10.1371/journal.pone.0221729)
Supplement: S1 File — (PDF) [file pone.0221729.s001.pdf]

Supporting information

S1 Structure of experiment system

In this paper, the structure of the experimental system is shown in (Fig A), (Fig A (a)) shows the direction of water flow and information transmission in the experimental system. (Fig A (b)) is the connection diagram of the main components in the experimental platform. The black arrow in (Fig A (a)) indicates the direction of flow passage. Water enters the pipeline from the water tank through the work of the gasoline pump. The pressure regulating valve is connected to adjust the pressure to collect experimental data under different pressures. The flow sensor is connected to the pipeline to measure the flow data in real time. The pressure sensor is installed in the pipeline to eliminate the pressure. The liquid pressure signal is obtained at the water inlet of the waterproof gun. In the initial state, the water gun is fixed on the platform parallel to the ground. In the experiment, the elevation angle and horizontal angle of the platform are adjusted to carry out the water gun jet experiment in different postures. The bottom of the platform is fixed on a highly adjustable lifting platform, and the height change of the water gun can be realized by adjusting the lifting platform. Hollow arrows indicate the direction of information transmission. PC can receive real-time signals collected by pressure, flow, wind speed, wind direction and temperature sensors by sending instructions. At the same time, PC can send instructions to set or adjust the angle of the platform. The elevating platform is tracted by a motor, and its height is adjusted by controlling the motor.

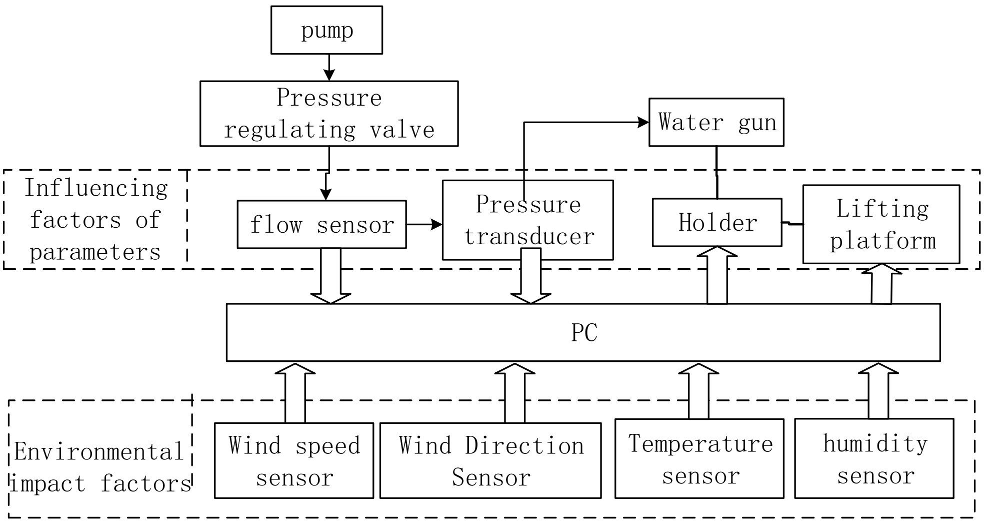

(a)

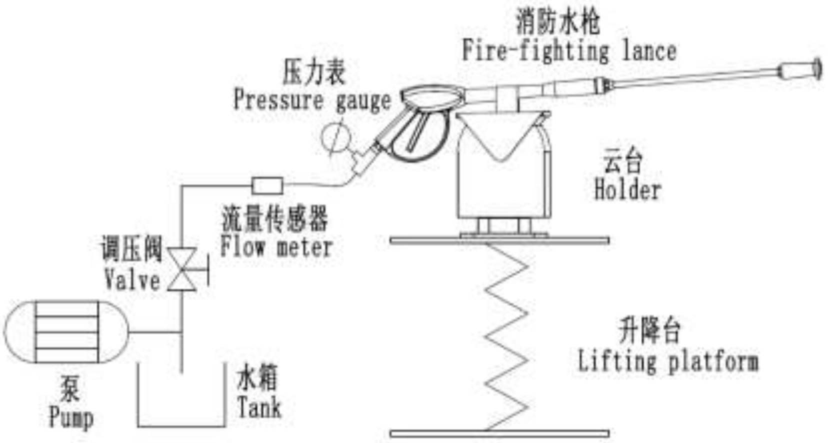

(b)

S1 Fig A Structure of experiment system

Pipeline connection and line connection of the experimental platform have been introduced in detail. The important equipment and instruments used in the experiment are introduced below.

(1) Flow Accumulator

In this experiment, the flow accumulator produced by Tianke Automation Instrument Co., Ltd. is selected. Its function is to collect the electrical signals obtained from the measurement of matching turbine flow sensor and pressure transmitter in real time, and complete the storage of these data.

(2) Turbine Flow Sensor

The flow sensor used in this experiment is a turbine type with a range of 0.2-1.2m^3/h. Its working principle is that when the fluid flows through the sensor, its impulse makes the turbine blade have rotating moment. The rotating speed of the impeller is proportional to the liquid velocity after the moment balance. The rotating blade cuts the magnetic force line so that the signal detector can output electrical signals. Flow sensor instructions require that the upstream and downstream end of the sensor should have a certain length of straight pipe section, and the liquid should be filled with the pipe without bubbles, so in this experiment, the upstream and downstream of the flow sensor are connected with a straight pipe and placed on the flat ground.

(3) Pressure sensor

Pressure sensor uses high-performance chip with a range of 0-1 MPa. It can convert the pressure change of liquid into 0-5 V voltage signal and output it to the integrator. In order to collect the pressure signal of the water gun inlet, the pressure sensor is installed at the joint of the water pipe and the water gun inlet through the tee.

(4) Holder

In order to change the angle of water gun in horizontal and vertical directions, the water gun is installed on the platform in this experiment. The change of water gun angle in both horizontal and vertical directions is realized by the rotation of the platform. The experiment adopts VT-L07 intelligent variable speed platform produced by Shandong Wanteng Electronics Co., Ltd. (see (Fig B)). The platform uses RS485 communication protocol, which can send instructions to adjust the angle in both horizontal and vertical directions, and can query the rotation angle in both directions in real time. Specific technical indicators are shown in (Tab A).

S1 Tab A Technical index of holder

Horizontal angle range/ °	Range of pitch angle/ °	Horizontal rotation speed/ °/s	Pitch rotation speed//s	positioning accuracy	interface	Input power supply /V
0-360	-45-70	0.01	0.01	0.1	RS485	DC 24

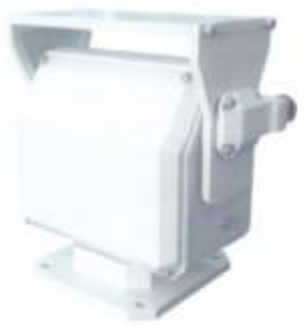

S1 Fig B VT-L07 Intelligent holder

(5) Elevator

In order to change the height of water gun, the platform was fixed on an elevator. The elevator can move upward and downward by controlling the positive and reverse rotation of DC motor. When it works, it can rise or fall at a uniform speed. The maximum load is about 200 kg, which satisfy the requirements of the experiment. The installation and construction of fire water gun, cloud platform and elevator are shown in (Fig C).

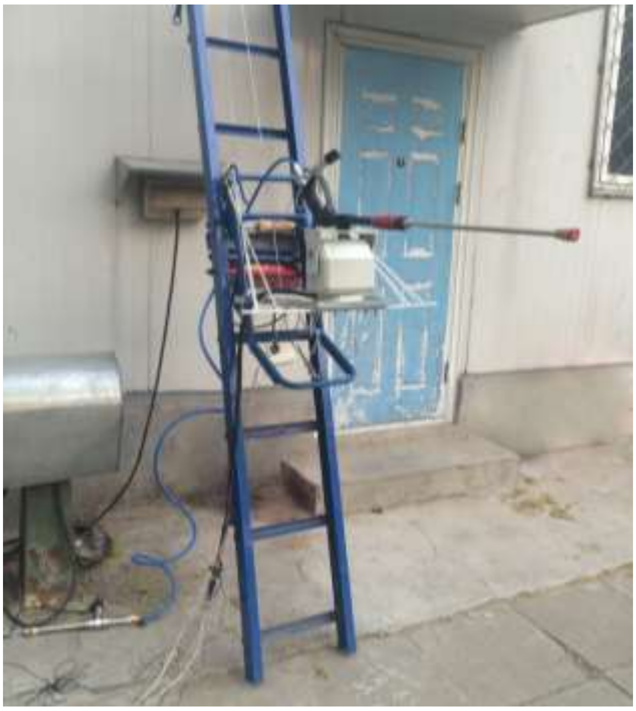

S1 Fig C Physical map of experiment platform

(6) Temperature, Wind Speed and Wind Direction Sensors

The wind speed is measured by a three-cup wind speed sensor (see (Fig D)). When the wind drives the wind cup to rotate, the integrated optoelectronic conversion mechanism inside the instrument can collect the rotation speed of the spindle and finally convert the wind speed. The wind direction sensor (see (Fig E)) is used to measure the wind direction, and the horizontal wind direction of 360 degrees can be measured. Wind speed and direction sensors are 0 ~ 5V voltage signal output. The output voltage signal of the wind direction sensor corresponds to the azimuth angle of 0-360 degree wind direction based on the North direction. The wind speed sensor can measure the wind speed in the range of 0-30m/s, and the output voltage signal corresponds to the wind speed range in equal proportion.

In this study, temperature, wind speed and wind direction sensors are integrated into an environmental monitoring device. See (Fig F). The environment sensors communicate with PC through RS232 protocol.

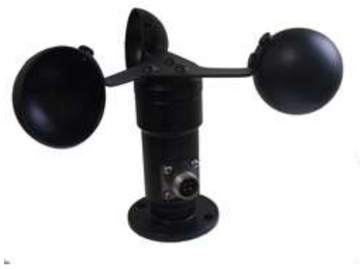

S1 Fig D Three-cup wind speed sensor

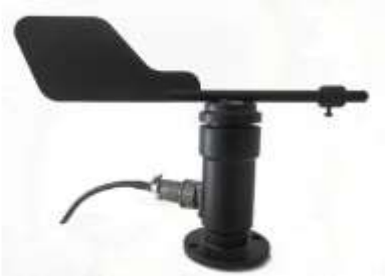

S1 Fig E Wind direction sensor

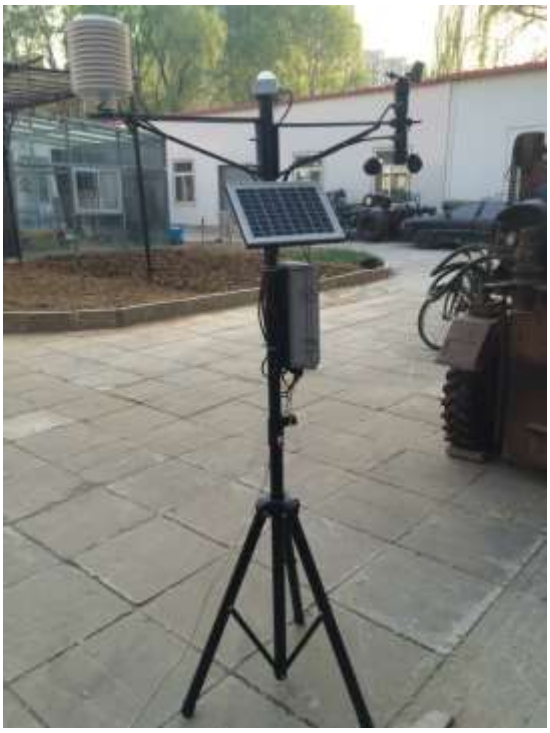

S1 Fig F Environmental monitoring device

References

1. Hao W R, Kan J M. Application of self-tuning fuzzy proportional–integral–derivative control in hydraulic crane control system[J]. Advances in Mechanical Engineering, 2016, 8(6): 1-10.

2. Dai Zhihui, Huang Qingqing, Li Wenbin, Zhang Chaoyi. Forest target classification research based on multi-class gauss kernel fuzzy support vector machine [J]. International Journal of Circuits, Systems and Signal Processing, 2017, 11: 52-58.
