# Supplementary material for: Prediction model for the water jet falling point in fire extinguishing based on a GA-BP neural network
Source: PLoS One. 2019 Sep 4;14(9):e0221729. doi: 10.1371/journal.pone.0221729 (PMC6726229; doi:10.1371/journal.pone.0221729)
Supplement: S3 File — (PDF) [file pone.0221729.s003.pdf]

Supporting information

S3 Platform experiment data of water jet system

Number of tests	Set the action brake	Pre-design Estimation Distance (cm)	Operational requirements （N）	Moving distance after remote control operation (cm)	Whether the gear is switched correctly
1		1.4	185.9		
2		1.2	193.8		
3		1.8	216.2		
4		1.7	216.7		
5		1.4	196.8		
6		1.6	177.3		
7		1.8	188.8		
8		2.0	194.1		
9		1.1	95.6		
10		1.2	107.4		
11		1.2	112.4		
12		1.3	111.3		
13		0.7	86.1		
14		0.8	82		
15		0.8	93.4		
16		0.6	85.1		
17		0.9	102.2		
18		1.0	103.4		

Number of tests	Set movement forward (throttle)	Estimating Distance of Drop-down Position before Design (cm)	Operational requirements （N）	Moving distance after remote control operation (cm)	Whether the gear is switched correctly
1		1.3	198		
		1.2	156		
		1.6	163.3		
		1.4	155.1		
		1.3	156.5		
		1.2	156.1		
		1.4	164.6		
		1.6	156.1		
		1.5	140.6		
		1.2	167.9		

		1.1	99.7		
		0.8	87.4		
		0.8	80.6		
		0.9	83		
		1.2	90.6		
		1.1	102.2		
		0.7	85.2		
		0.6	84.6		
		0.9	97.4		
		0.8	100.5		
		0.6	84.8		
		1.1	103.7		
		1.5	140.6		
		1.4	152.9		
		1.6	163.5		
		1.2	126.9		

Number of tests	Setting Action Shift-High Speed	Designing the front baffle to move forward to measure distance (cm)	Operational requirements （N）	Moving distance after remote control operation (cm)	Whether the gear is switched correctly
1		2.6	54		
		2	62		
		2.4	41.8		
		2.6	42.8		
		2.7	42.6		
		2.6	54		
		2.8	37.5		
		2.8	48.3		
		2.5	48.6		

Number of tests	Set Action Shift-Forward-Right	Pre-design Estimation Distance (cm)	Operational requirements （N）	Moving distance after remote control operation (cm)	Whether the gear is switched correctly
1		2.6	32.2		
		1.9	28.1		
		2.2	27.5		
		2.2	31.1		
		2.5	29.3		
		2.4	29.3		

		2.2	24.1		
		1.9	25.4		
		2.1	25.4		

Number of tests	Setting Action	Estimating Distance by Designing Front Bar(cm)	Operational requirements （N）	Moving distance after remote control operation (cm)	Whether the gear is switched correctly
1	Shift-Forward-Forward	3.2	75.2		
		3.6	70.5		
		3.5	71.8		
		4	74.9		
		4	76.5		
		4	73.6		
		4	75.5		
		3.6	67.5		
		3.5	65.3		

Number of tests	Set Action Shift-Back Off	Estimation of Distance by Designing Front Bar (cm)	Operational requirements （N）	Moving distance after remote control operation (cm)	Whether the gear is switched correctly
1		2.2	68.5		
		2.0	68.7		
		1.7	67.2		
		1.7	70.4		
		2.4	64.2		
		2.1	82.1		
		1.6	85.3		
		2	66.1		
		2.1	89.3		
		2.3	64.8		
